# Endogenous Estrogen-Mediated Heme Oxygenase Regulation in Experimental Menopause

**DOI:** 10.1155/2015/429713

**Published:** 2015-05-06

**Authors:** Anikó Pósa, Renáta Szabó, Anett Csonka, Médea Veszelka, Anikó Magyariné Berkó, Zoltán Baráth, Rudolf Ménesi, Imre Pávó, Mariann Gyöngyösi, Ferenc László, Krisztina Kupai, Csaba Varga

**Affiliations:** ^1^Department of Physiology, Anatomy and Neuroscience, Faculty of Science and Informatics, University of Szeged, Kozep Fasor 52, Szeged 6726, Hungary; ^2^Faculty of Dentistry and Department of Orthodontics and Pediatric Dentistry, University of Szeged, Szeged 6720, Hungary; ^3^Department of Cardiology, Medical University of Vienna, Waehringer Guertel 18-20, 1090 Vienna, Austria

## Abstract

Estrogen deficiency is one of the main causes of age-associated diseases in the cardiovascular system. Female Wistar rats were divided into four experimental groups: pharmacologically ovariectomized, surgically ovariectomized, and 24-month-old intact aging animals were compared with a control group. The activity and expression of heme oxygenases (HO) in the cardiac left ventricle, the concentrations of cardiac interleukin-6 (IL-6) and tumor necrosis factor-*α* (TNF-*α*), the myeloperoxidase (MPO) activity in the cardiac left ventricle, and the effects of heme oxygenase blockade (by 24-hour and 1-hour pretreatment with tin-protoporphyrin IX, SnPP) on the epinephrine and phentolamine-induced electrocardiogram ST segment changes *in vivo* were investigated. The cardiac HO activity and the expression of HO-1 and HO-2 were significantly decreased in the aged rats and after ovariectomy. Estrogen depletion was accompanied by significant increases in the expression of IL-6 and TNF-*α*. The aged and ovariectomized animals exhibited a significantly elevated MPO activity and a significant ST segment depression. After pretreatment with SnPP augmented ST segment changes were determined. These findings demonstrate that the sensitivity to cardiac ischemia in estrogen depletion models is associated with suppression of the activity and expression of the HO system and increases in the secretion of proinflammatory cytokines and biomarkers.

## 1. Introduction

Many epidemiological studies have suggested the involvement of free radicals and oxidative stress in aging and certain age-related processes that often accompany the menopause [1–3]. An increased level of production of reactive oxygen species (ROS) is considered to be one of the major causes of age-related morbidity (e.g., coronary artery and general cardiovascular dysfunctions) [4]. Estrogen protects women against cardiovascular diseases and seems to play a major role in sex-related differences in hypertension in experimental models [5]. Antioxidant properties may also be involved since estradiol can reduce the expression of nicotinamide adenine dinucleotide phosphate (NADPH) oxidase subunits and increase the expression of superoxide dismutase [6, 7].

Estradiol protects endothelial cells against damage by oxidants and induces the generation of endothelial derived vasodilators, such as nitric oxide (NO) [8]. Recent data indicate that another system associated with cardioprotection, the heme oxygenase (HO) system, is also affected by estrogen [9]. HO-1 can catalyze the oxidative degradation of heme to yield equimolar amounts of biliverdin, free iron, and carbon monoxide (CO). Biliverdin is metabolized to bilirubin by biliverdin reductase. Among the products of HO-1, bilirubin and biliverdin are the most potent endogenous scavengers of ROS and CO exerts antiapoptotic and anti-inflammatory effects through the induction of soluble guanyl cyclase. CO additionally suppresses the production of proinflammatory cytokines, such as tumor necrosis factor-alpha (TNF-*α*) [10].

The aim of our work was to investigate the effects of estrogen depletion on the activation and expression of HO in the cardiovascular system and on inflammatory biomarkers, such as the levels of TNF-*α* and interleukin-6 (IL-6) and the activity of myeloperoxidase (MPO) in experimental menopause.

## 2. Methods

### 2.1. Experimental Groups of Animals

All experimental procedures were performed in accordance with the standards of the European Community guidelines on the care and use of laboratory animals and had been approved by the Institutional Ethics Committee.

Female Wistar rats were divided into four groups: 4-month-old sham-operated controls, 4-month-old pharmacologically ovariectomized (POVX) animals, 4-month-old surgically ovariectomized (OVX) animals, and 24-month-old ovary-intact (aged) animals.

Ovariectomy surgery was performed on anesthetized animals via a small ventral abdominal midline incision. The ovaries were clamped bilaterally and removed. The uterine horns were tied and the uterus was left intact. The abdominal wall was then sutured. In the sham procedure, the animals were anesthetized and the abdominal wall was opened but the ovaries were not removed. The ovaries were exteriorized to create a similar degree of stress. Other groups of female rats received treatment with 750 *μ*g/kg triptorelin (Decapeptyl depot, Ferring, Germany) i.m. every 4 weeks in order to achieve pharmacological ovariectomy [11]. After a 6-week resting period to verify the surgically or pharmacologically induced menopause and to ensure that all animals were killed at the same stage of the estrus phase (all the control rats were in the proestrus phase) via Giemsa staining method we checked the estrogen level with estrogen quantitative enzyme-linked immunosorbent assay according to the manufacturer's directions (Quantikine rat Estrogen Elisa kit, R&D Systems Inc.) [12].

### 2.2. Cardiac Left Ventricle HO Activity

The cardiac left ventricle (LV) tissues were homogenized (Ultra Turrax T25; 13.500 min^−1^; 2 × 30 s) in ice-cold 10.0 mM N-2-hydroxyethylpiperazine-N′-2-ethanesulfonic acid, 32.0 mM sucrose, 1.0 mM dithiotreitol, 0.10 mM ethylenediaminetetraacetic acid disodium salt dihydrate (EDTA), 10.0 *μ*g/mL trypsin inhibitor, 10.0 *μ*g/mL leupeptin, and 2.0 *μ*g/mL aprotinin; pH 7.4. The supernatant was collected by centrifugation at 15000 g for 20 min at 4°C. The reaction mixture contained the following compounds in a final volume of 1.50 mL: 2.0 mM glucose-6-phosphate, 0.14 U/mL glucose-6-phosphate dehydrogenase, 15.0 *μ*M hemin, 120.0 *μ*g/mL rat liver cytosol as a source of biliverdin reductase, 2.0 mM MgCl_2_  × 6H_2_O, 100.0 mM KH_2_PO_4_, and 150.0 *μ*L of supernatant. To start the reaction 100.0 *μ*L the reduced form of *β*-nicotinamide adenine dinucleotide phosphate, reduced form (150.0 *μ*M), was added to the samples and they were then incubated in the dark at 37°C for 60 min. The reaction was stopped by placing the samples on ice. The bilirubin formed was calculated from the difference between the optical densities obtained at 464 and 530 nm. Bilirubin solution was used as standard (58.47 *μ*g/mL; 10.0 *μ*M). Protein content was determined by spectrophotometric assay (Bio-Rad Protein Assay).

One unit of HO activity was defined as the amount of bilirubin (nmol) produced per hour per mg protein.

### 2.3. Cardiac LV HO-1 and HO-2 Expression

The expression of HO-1 and HO-2 enzymes was determined by Western blot analysis. The cardiac LV tissues were homogenized (Ultra Turrax T25; 13.500 min^−1^; 2 × 30 s) in ice-cold Tris-mannitol buffer (2.0 mM Tris 7–9, 50.0 mM mannitol, 100.0 *μ*M phenyl methyl sulfonyl fluoride, 2.0 *μ*M leupeptin, 0.50 mU/mL aprotinin, and 0.50% Triton X-100). Homogenizates were centrifuged at 12000 g for 20 min at 4°C. Protein content was measured by spectrophotometric assay (Bio-Rad Protein Assay).

Aliquots of 25.0 *μ*g of total cellular protein were denatured by mixing and boiling with 20.0 mM Tris 7–9, 3.0 mM EDTA, 2.0% sodium dodecyl sulfate, 10.0% *β*-mercaptoethanol, and 20.0% glycerol. The samples were electrophoresed (100 V, 50 mA) on 10.0% polyacrylamide gel and transferred (100 V, 100 mA, 2 h) to a nitrocellulose membrane (Amersham, Pharmacia Biotech., Buckinghamshire, UK). Equal protein loading was determined by staining the blot with 0.10% Ponceau red in 5.0% acetic acid. Two h after blocking with PBS (pH 7.4), 0.25% Tween 20, and 5.0% fat-free dried milk, the membrane was probed for 2 h with mouse anti-HO-1 monoclonal antibody (1/10.000; StressGen Biotechnologies Corp., Victoria, Canada) or anti-HO-2 monoclonal antibody (1/1000; StressGen Biotechnologies Corp., Victoria, Canada) at room temperature, washed 3 times with PBS-Tween 20, and then incubated for 1 h with horseradish peroxidase-conjugated bovine anti-mouse antibody (1/2000; Santa Cruz Biotechnology Inc., Santa Cruz, CA, USA) at room temperature. Membranes (Hybond ECL Nitrocellulose membrane, Amersham, Pharmacia Biotech., Buckinghamshire, UK) were developed by using an enhanced chemiluminescence system (ECL+Plus, Amersham Pharmacia Biotech., Buckinghamshire, UK) and exposed to Hyperfilm (Biomax light-1, Eastman Kodak Comp., Rochester, NY, USA). Films were analyzed by using ImageQuant Software (Amersham Pharmacia Biotech., Buckinghamshire, UK) after scanning with GelAnalyst 3.01 Software (Iconix, Toronto, Canada).

### 2.4. Measurement of Cardiac LV IL-6 and TNF-*α* Concentrations

The IL-6 and TNF-*α* levels were determined by means of quantitative enzyme-linked immunosorbent assays according to the manufacturer's directions (Quantikine rat IL-6 and TNF-alpha Elisa kit, R&D Systems Inc.). Optical density was measured at 450 nm (Benchmark Microplate reader; Bio-Rad). IL-6 and TNF-*α* were expressed as pg/mg protein.

### 2.5. Cardiac LV MPO Activity

The cardiac LV tissues were homogenized (Ultra Turrax, T25, 13 500 r.p.m., twice for 30 s) in ice-cold phosphate buffer (50 mM, pH 6.0), freeze-thawed three times, and then centrifuged twice (each time at 15 000 g for 15 min at 4°C). A 12 *μ*L aliquot of the supernatant was next mixed with 280 *μ*L of phosphate buffer (50 mM, pH 6) containing 0.167 mg mL^−1^ of O-dianisidine dihydrochloride and the reaction was started with 10 *μ*L of 0.03% hydrogen peroxide and assayed spectrophotometrically at 490 nm (Benchmark Microplate reader; Bio-Rad, Budapest, Hungary) after 90 s of shaking. MPO activity was expressed as mU/mg protein.

### 2.6. Experimental Angina Provoked by Epinephrine Plus Phentolamine

The standard limb lead II of the surface electrocardiogram (ECG) was recorded by the HAEMOSYS system (Experimetria Ltd., Budapest, Hungary). The change in the ST segment was measured and used as the index of angina severity. The mean ECG voltage 13 ms after the peak of the S wave was defined as the value of the ST segment, as described previously [13, 14]. The difference in the amplitude of the ST segment after and before the administration of the angina-provoking agents was calculated and expressed as the depression of the ST segment in mV. In the epinephrine plus phentolamine model, a single dose of epinephrine (10.0 *μ*g/kg) and 30 s later the *α*-adrenoceptor antagonist phentolamine (15.0 mg/kg) were administered into the tail vein of the rat. Each agent was dissolved in 0.20 mL of physiological saline and injected over 2 s. The ECG was recorded simultaneously. To investigate the effects of HO enzyme activity inhibition on ST segment changes tin-protoporphyrin IX (30.0 *μ*mol/kg, s.c., pH 7.4) was administrated 24 h and 1 h before treatment.

### 2.7. Chemicals

Apart from epinephrine (Tonogen, Richter Gedeon, Hungary), Phentolamine (Regitin, P; Ciba-Geigy, Switzerland), SnPP (Frontier Scientific Europe, United Kingdom), and triptorelin (Decapeptyl depot, Ferring, Germany), all chemicals were from Sigma Aldrich Company.

### 2.8. Statistical Analysis

Data are reported as means ± S.E.M. of the results from at least 3 independent experiments. Western blots are shown as representative photographs of three independent experiments. Statistical significance was assessed by ANOVA; *P* < 0.05 was taken as significant.

## 3. Results

### 3.1. Cardiac LV HO Activity

**Figure 1:**
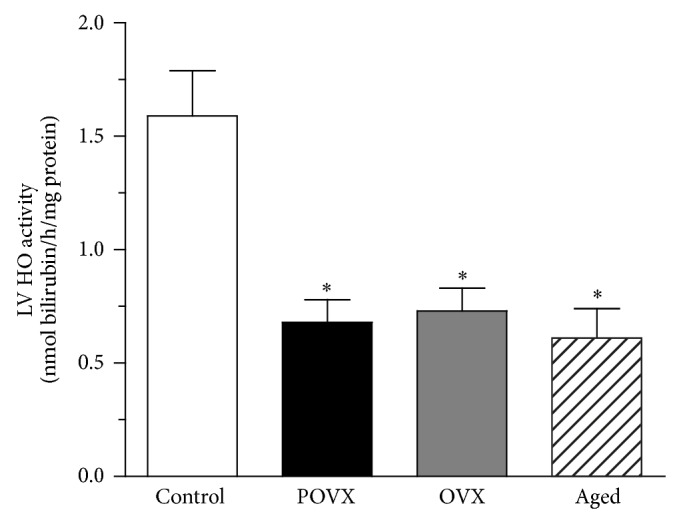
Heme oxygenase activity (HO; expressed as nmol/bilirubin/h/mg protein) in the cardiac left ventricle of control, ovariectomized (pharmacologically POVX and surgically OVX), and aged rats. A significant decrease was observed in the HO activity of the ovariectomized and aged rats as compared with the controls. Data are expressed as means ± S.E.M., *n* = 11-12. Statistical significance: ^*^ *P* < 0.05 relative to the control (sham-operated) group.

As shown in Figure 1, the activity of HO was determined by measurement of bilirubin formation in the cardiac LV. We found that HO activity was significantly (^*^ *P* < 0.05) decreased in the ovariectomized (POVX: 0.68 ± 0.1 nmol bilirubin/h/mg protein, *n* = 12; OVX: 0.73 ± 0.1 nmol bilirubin/h/mg protein, *n* = 11) and aged (0.61 ± 0.13 nmol bilirubin/h/mg protein, *n* = 11) animals compared to the control group (1.59 ± 0.2 nmol bilirubin/h/mg protein, *n* = 11).

### 3.2. Cardiac LV HO-1 and HO-2 Expression

**Figure 2:**
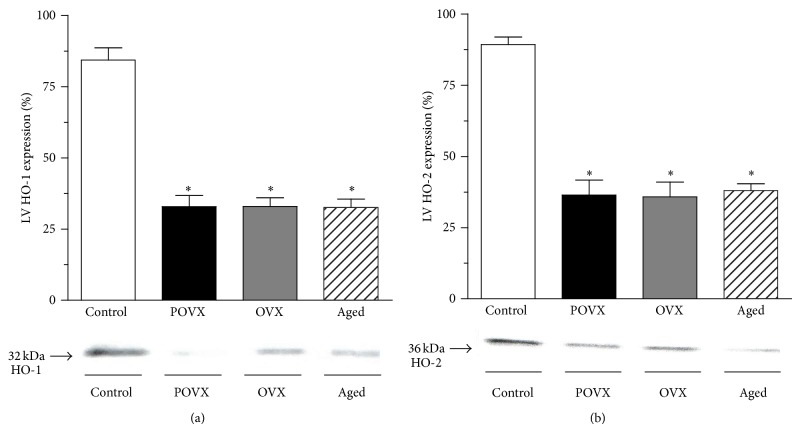
Detection of heme oxygenase expression (HO-1 and HO-2; expressed as %) by Western blot analysis in the cardiac left ventricle of control, ovariectomized (pharmacologically POVX and surgically OVX), and aged rats. (a) Heme oxygenase-1 enzyme (HO-1; expressed as %, 100% being the maximal expression) in the cardiac left ventricle of control, ovariectomized (pharmacologically POVX and surgically OVX), and aged rats with densitometric assessment. There was a significant decrease in HO-1 expression in the ovariectomized and aged groups. Data are expressed as means ± S.E.M., *n* = 10. Statistical significance: ^*^ *P* < 0.05 relative to the control (sham-operated) group. (b) Heme oxygenase-2 expression (HO-2; expressed as %, 100% being the maximal expression) in the cardiac left ventricle of control, ovariectomized (pharmacologically POVX and surgically OVX), and aged rats, with densitometric assessment. The HO-2 expression proved to be significantly decreased in the ovariectomized and aged rats. Data are expressed as means ± S.E.M., *n* = 10. Statistical significance: ^*^ *P* < 0.05 relative to the control (sham-operated) group.

HO-1 and HO-2 protein were determined by Western blot techniques. Data are shown in Figure 2. In both the POVX and OVX rats, the cardiac expression of HO-1 (POVX: 32.87 ± 3.92%, *n* = 10; OVX: 32.92 ± 3.1%, *n* = 10) and HO-2 (POVX: 36.4 ± 5.3%, *n* = 10; OVX: 35.8 ± 5.22%, *n* = 10) was significantly (^*^ *P* < 0.05) lower than in the controls (HO-1: 84.33 ± 4.3%, *n* = 10; HO-2: 89.29 ± 2.6%, *n* = 10). Aging was also accompanied by a significantly (^*^ *P* < 0.05) reduced cardiac expression of HO-1 (32.62 ± 2.89%, *n* = 10) and HO-2 protein (37.95 ± 2.42%, *n* = 10) relative to the control group.

### 3.3. Cardiac LV IL-6 and TNF-*α* Concentrations

**Figure 3:**
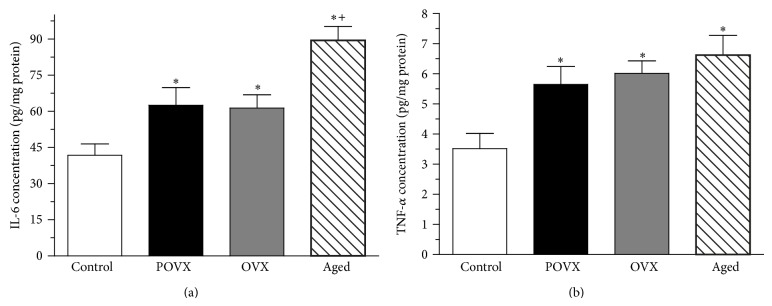
The levels of the proinflammatory cytokines interleukin- (IL-) 6 and tumor necrosis factor-alpha (TNF-*α*) in control, ovariectomized (pharmacologically POVX and surgically OVX), and aged rats. (a) Cardiac LV IL-6 level (expressed as pg/mg protein) in control, ovariectomized (POVX, OVX), and aged rats. The IL-6 level was significantly increased in the ovariectomized rats as compared with the control. The aged group exhibited a more significant increase than that of the ovariectomized group. Data are shown as means ± S.E.M., *n* = 8–10. Statistical significance: ^*^ *P* < 0.05 relative to the control group, and ^+^ *P* < 0.05 relative to the ovariectomized animals. (b) Cardiac LV tumor necrosis factor-alpha concentration (TNF-*α*; expressed as pg/mg protein) in control, ovariectomized (POVX, OVX), and aged rats. The TNF-*α* concentration was significantly higher in the ovariectomized and aged animals. Data are expressed as means ± S.E.M., *n* = 6–8. Statistical significance: ^*^ *P* < 0.05 relative to the control (sham-operated) group.

The levels of the proinflammatory cytokines IL-6 and TNF-*α* were significantly (^*^ *P* < 0.05) elevated during aging and after ovariectomy in both the POVX and OVX groups in comparison with the control animals. Additionally, the increase in cardiac LV IL-6 concentration was more marked in the aged group than in the ovariectomized and control animals (IL-6 concentration in the aged group: 89.434 ± 5.817 pg/mg protein, *n* = 8; in the POVX group: 62.503 ± 7.339, *n* = 9; in the OVX group: 61.403 ± 5.512 pg/mg protein, *n* = 8; and in the controls: 41.797 ± 4.673 pg/mg protein, *n* = 9; TNF-*α* concentration in the aged group: 6.622 ± 0.657 pg/mg protein, *n* = 8; in the POVX group: 5.648 ± 0.598 pg/mg protein, *n* = 8; in the OVX group: 6.015 ± 0.415 pg/mg protein, *n* = 6; and in the controls: 3.520 ± 0.502 pg/mg protein, *n* = 7). The results are presented in Figure 3.

### 3.4. Cardiac LV MPO Activity

**Figure 4:**
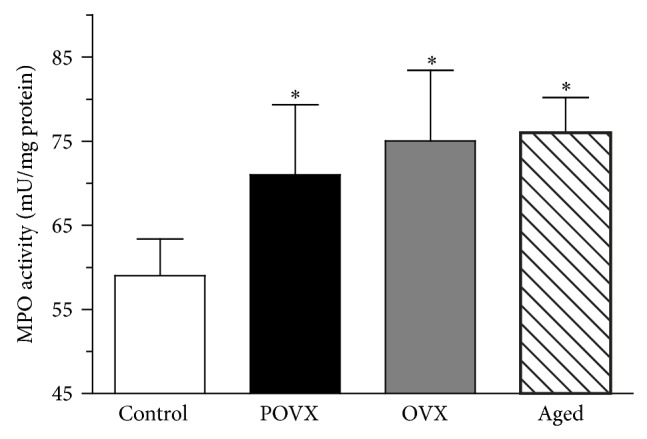
Myeloperoxidase activity (MPO; expressed as mU/mg protein) in the myocardium of control, ovariectomized (pharmacologically POVX, surgically OVX), and aged rats. The MPO enzyme activity was significantly enhanced in the ovariectomized and aged animals. Results are shown as means ± S.E.M., *n* = 7-8. Statistical significance: ^*^ *P* < 0.05 relative to the control (sham-operated) group.

The activity of MPO was significantly (^*^ *P* < 0.05) higher in the cardiac LV of the POVX (71.0 ± 8.34 mU/mg protein, *n* = 7), OVX (75.0 ± 8.42 mU/mg protein, *n* = 8), and aged (76.0 ± 4.192 mU/mg protein; *n* = 8) groups as compared to control ones (59.00 ± 4.367 mU/mg protein, *n* = 8). The data are to be seen in Figure 4.

### 3.5. Effects of Inhibition of HO Activity on Cardiac Ischemia

**Figure 5:**
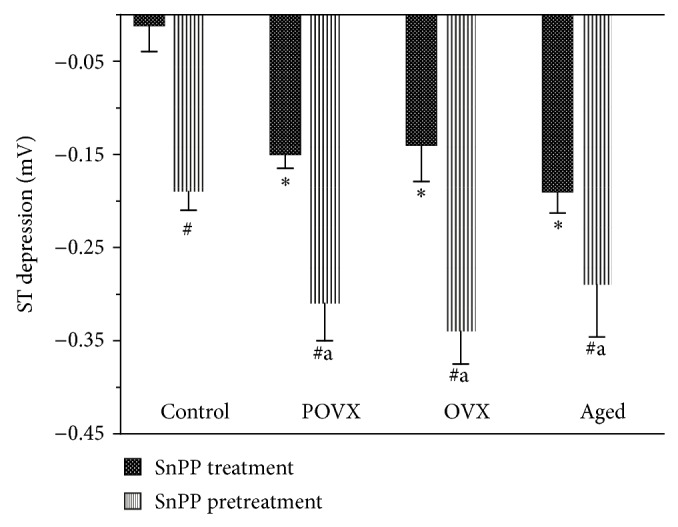
The effects of HO activity inhibition by tin-protoporphyrin-IX (SnPP) on the ST segment (measured in a lead II standard surface ECG, expressed in mV) following intravenous injection of epinephrine (10.0 *μ*g/kg) and 30 s later phentolamine (15.0 mg/kg). The columns show significant decreases in the ST segment of ovariectomized and aged animals without SnPP treatment. SnPP pretreatment (30.0 *μ*mol/kg, s.c., pH 7.4, 24 h and 1 h pretreatment) caused a significant ST depression in the sham-operated control animals. Following ovariectomy (POVX and OVX), or in aged rats, the SnPP treatment induced a more marked significant decrease in ST as compared with the control. Results are shown as means ± S.E.M., *n* = 11–13. Statistical significance: ^*^ *P* < 0.05 relative to the ovary-intact sham-operated control group; ^#^ *P* < 0.05 a significant difference between groups with and without SnPP pretreatment; ^a^ *P* < 0.05 a significant difference between the data for the ovariectomized and aged groups after SnPP pretreatment and those for the controls.

The administration of phentolamine caused a significant (^*^ *P* < 0.05) ST segment depression 30 s following epinephrine administration in the POVX (−0.15 ± 0.015 mV, *n* = 13), OVX (−0.14 ± 0.0389 mV, *n* = 13), and aged (−0.19 ± 0.023 mV, *n* = 13) rats. In the ovary-intact sham-operated females, the ST segment depression was −0.0116 ± 0.028 mV, *n* = 4. Pretreatment with the HO inhibitor SnPP (30.0 *μ*mol/kg, s.c. pretreatment 24 h and 1 h prior to the measurement) caused an ST depression in the sham-operated animals (−0.19 ± 0.02 mV, *n* = 11), and significantly (^#^ *P* < 0.05) augmented the ST depression in the POVX (−0.31 ± 0.04 mV, *n* = 3), OVX (−0.34 ± 0.035 mV, *n* = 11), and aged (−0.29 ± 0.056 mV, *n* = 11) groups. The data are depicted in Figure 5.

## 4. Discussion

We conclude that pharmacological treatment with an aromatase inhibitor triptorelin (POVX) and surgical ovariectomy (OVX) are valuable conditions for effective estrogen studies.

The female cardiovascular system is influenced by changes in the endocrine system. During the menopausal transition, dramatic hormonal changes such as declining levels of estrogen and rising levels of gonadotropins may affect the cardiovascular system. Estrogen deficiency induces an imbalance between enhanced ROS production and inadequate antioxidant activity. The decline in ovarian function accompanying the menopause, OVX, and POVX contributes to the induction of proinflammatory cytokines such as TNF-*α* and IL-6. Estrogen is a well-known regulator of inflammation. Cardiovascular and immune system abnormalities have been reported in females with estrogen deficiency, exerting a number of known anti-inflammatory effects through a variety of different mechanisms, both genomic and nongenomic. The anti-inflammatory role ranges from generating NO and regulating leukocyte recruitment to reducing oxidative stress and promoting cell survival. These effects contribute to dampening inflammation in the vascular system. Estrogen deficiency has been shown to upregulate TNF-*α* levels in aged animals with deleterious effects on vascular function. Such effects are largely mediated through increased oxidative stress and can be reversed through exogenous estrogen, a TNF-*α* inhibitor, or antioxidants [15]. Sex hormones are known modifiers of the inflammatory response to injury, an important aspect of myocardial dysfunction and cardiomyocyte death following ischemia. Hamilton et al. in their experiments showed that the overall effect of OVX on myocardial gene expression was increased expression of genes involved in the inflammatory response. OVX increased IL-6 receptor, TNF-*α*, complement 8, and SOCS2 and SOCS3 expression [16]. During aging and after ovariectomy, estrogen deficiency is able to stimulate the spontaneous secretion of such proinflammatory cytokines. Along with the effects of cytokines, the MPO activity is determined as an inflammatory biomarker. Inflammation and oxidative stress are associated with atherosclerosis and cardiovascular disease. Conversely, inflammation also triggers vascular remodeling, aggravates vessel injury, and exacerbates the processes of hypertension and atherosclerosis [17]. MPO plays a significant role in the development of the atherosclerotic lesion and renders the plaques unstable, which is associated with the aging mechanism and cardiovascular disease [18]. Similar results demonstrate the role of HO-1 in HO-1 knockout mice, in which the HO-1 deficiency leads to an increased production of proinflammatory cytokines [9] while HO-1 upregulation successfully slows the processes of hypertension and myocardial infarction [19]. Preclinical and clinical evidence clearly suggest that the progression of atherosclerosis is associated with inflammation. The CO derived from HO has been demonstrated to protect against TNF-*α*-induced apoptosis [20]. Aging leads to significantly increased levels of the proinflammatory cytokines in the liver of aged female rats as compared with young controls. Hence, the diminished activity of the HO system contributes to increased oxidative stress [21]. Ross and Howlett demonstrated that the ventricular myocytes from young female rats were more resistant to ischemia and reperfusion injury than were cells from males, and then ischemia and reperfusion injury in female myocytes was exacerbated by aging or by OVX. Advancing age abolishes the beneficial effects of the female sex on the cell viability and the contractile function [22]. Our data indicated that aging and estrogen depletion process abolished the beneficial effects of the female sex in the myocardium. This suggests that the cardioprotection of the female sex may decline with age in response to a reduction in circulating estradiol levels. Indeed, we found that the removal of estrogen through OVX or POVX exacerbated the adverse effects of ischemia, which can be augmented via HO activity inhibition. Our results indicate that decreases in the expression of HO-1/HO-2 and in the activity of the HO system are important factors that contribute to the enhanced sensitivity of the OVX or POVX heart to ischemia. Moreover, we found that aging and OVX induced diminished HO activity and expression. Direct vascular effects of estradiol are believed to play a significant role in the cardioprotectivity of estrogens. These findings on OVX and POVX female rats may have important clinical implications. Extensive epidemiological studies of the morbidity and mortality rates of postmenopausal women have revealed a significant increase in cardiovascular mortality as compared with that in women who are still menstruating. After the menopause, women lose the relative cardiovascular protection that they had previously enjoyed over men [23, 24]. Although estrogen has been reported to protect the cardiovascular system, the mechanisms involved remain unclear. Estrogen exerts genomic and nongenomic effects via estrogen receptor-dependent and receptor-independent mechanisms, to confer protective effects on the cardiovascular system. Alterations in plasma concentrations of lipoproteins (decreases in low-density lipoprotein levels and in oxidized low-density lipoprotein formation and increases in high-density lipoprotein levels), hemostatic factors, glucose, insulin, and endothelium-derived factors (decreases in endothelin and increases in NO and prostaglandins) and the inhibition of smooth muscle cell migration and proliferation induced by various mitogens are thought to contribute to the vasoprotective effects of estrogen [25]. Of these biological effects, the antioxidant effects of estrogen may play a critical role in eliciting vasoprotective effects [26]. Endogenous and exogenous estrogens have antioxidant potential both* in vitro* [27] and* ex vivo* [28]. An estrogen deficiency in the menopausal state therefore leads to changes in the homeostatic environment of the body, for example, a gradual increase in oxidative stress. A previous report from our laboratory supports this concept, demonstrating the sexual dimorphism in HO activity and expression. The present study demonstrated similar results in that the HO levels in the hearts of OVX rats were markedly lower than those in the sham-operated controls. HO has potent cytoprotective effects that are likely to be mediated by its products, CO, biliverdin/bilirubin, and free iron, and we were interested in the role of HO in an estrogen-deficient environment. The HO levels proved to be reduced in the aged and ovariectomized rats, which might result from the downregulation of HO in the heart tissues. After OVX, a marked reduction in estrogen synthesis led to decreases in the activity and expression of HO.

We also found that the cardiac LV levels of IL-6 and TNF-*α* were significantly reduced after ovariectomy and the menopause. An estrogen deficiency may lead to an increased activity of MPO.

However, only a small amount of data is available concerning the effects of HO expression* in vivo*. It emerged from the current study that an HO activity inhibitor caused an ST depression in control female rats and augmented the ST depression in the estrogen-withdrawal model. The data suggest that the HO activity protects the heart at a fertile age.

This study has also demonstrated that the inhibition of HO activity in ovariectomized rats significantly increases the ST depression, implying that HO plays a protective role in the cardiovascular system. Although a number of studies have clearly pointed to the vasculoprotective effects of estrogen in animal models, the targets of estrogen are numerous and estrogen deficiency affects the cardiovascular system at different points. In summary, the decreased level and activity of HO in the hearts of OVX and POVX rats may cause the development of atherosclerosis after the menopause.
